# What Happens at a Dental Surgery When the Patient is a Child with Autism Spectrum Disorder? An Italian Study

**DOI:** 10.1007/s10803-020-04684-0

**Published:** 2020-09-03

**Authors:** Maria Grazia Mada Logrieco, Giuseppe Niccolò Ciuffreda, Bruna Sinjari, Maria Spinelli, Rodrigo Rossi, Gianmaria D’Addazio, Francesca Lionetti, Sergio Caputi, Mirco Fasolo

**Affiliations:** 1 Department of Neuroscience Imaging and Clinical Sciences, University “G. D’Annunzio” Chieti-Pescara, Via dei Vestini 33, 66100 Chieti, CH Italy; 2 Department of Economics, University of Pisa, Pisa, Italy; 3 Department of Medical Oral and Biotechnological Sciences, University “G. D’Annunzio” Chieti-Pescara, Via dei Vestini 31, 66100 Chieti, CH Italy

**Keywords:** Autism, Oral health care, Children, Typical development children, Parents, Dentist

## Abstract

Oral health care can be a difficult experience for a child with Autism Spectrum Disorder (ASD), for their family and for the dentist. The purpose of this study is to provide an understanding of the challenges experienced by the three aforementioned figures during oral care treatment. A cohort of 275 parents of typical development children (TD), 57 parents of children with ASD (3–15 years old) and by 61 dentists, completed two different multiple choices questionnaires. The data obtained show a great difficulty in the treatment of children with ASD as seen by the dentists and by the parents. This is due to: caregivers’ demographic issues; difficulties encountered before and during the dental examination; scarce presence of experts in ASD treatment.

Oral care is an important component of health care. Inadequate oral care and the illness that can result from it can have a negative effect on health and quality of life (Casamassimo 1996; Hulland and Sigal 2000). Despite the importance of oral care, the disparities between children with special health care and typical development (TD) children still exist, in the access and practice of oral care, with dental care being the most frequently cited unmet health care need (Lewis et al. 2005). One specific and wide subgroup of these patients, are patients with Autism Spectrum Disorder (ASD). Worldwide prevalence of autism is just under 1%, but estimates are higher in high income countries. This is mainly due to increasing awareness of diagnostic criteria, which affects the reported prevalence in high income countries and limited datasets in low income countries (Lord et al. 2020).

ASD is a set of neurodevelopmental conditions, some of which can be attributed to distinct etiological factors. The majority are probably the results of complex interactions between genetic and non-genetic risk factors. The latest revision of DSM American Psychiatric Association Diagnostic and Statistical Manual of Mental Disorders. 5th edn. (APA 2013), adopted the umbrella term Autism Spectrum Disorder with two features: difficulties in social communication and social interaction; and restricted and repetitive behavior, interests or activities.

Problems with communication appear in many ways among these patients. Almost half of children with ASD are completely nonverbal (Rose et al. 2016). Some of them do not make any gesture to compensate verbal communication (Lai et al. 2014), others present a stereotyped or echolalic language (Johnson and Myers 2007).

Children with ASD can also present impairment of social interactions, such as unusual eye-contact, limitations in facial expressions directed towards other people, atypical social engagement and responsiveness, difficulty with peer relationships, lack of awareness or understanding of other people’s thoughts and feelings, poor communication skills, difficulty initiating social contacts through verbal or non-verbal means, rigid or unusual behaviors and restricted interests (Lai et al. 2014).

One more crucial symptom is the higher sensory sensitivities presented by children with ASD (Stein et al. 2012). This aspect is present in almost 95% of the affected population (Ben Sasson et al. 2009; Tomcheck and Dunn 2007).

These sensory sensitivities include tactile, gustatory, auditory, visual, and vestibular stimuli. Another important aspect of ASD symptomatology is the pattern of restricted and repetitive behaviors.

These two sets of symptoms have a wide range of severity levels, which may be different for each patient with ASD (Johnson and Myers 2007). However, these characterised symptoms of ASD can have a deep impact on the way dentists can provide dental treatment for these patients.

Most research indicates that children with ASD exhibit a high prevalence of poor oral health, as assessed by prevalence and severity of caries (De Mattei et al. 2007; Jaber 2011; Kopycka-Kedzierawski and Auinger 2008; Marshall et al. 2010), despite a small group of contrary studies (Fahlvik-Planefeldt and Herrstrom 2001; Loo et al. 2008; Morinushi et al. 2001). Although not a direct cause of dental deficit, life factors and behaviors prevalent in ASD patients are associated with increased caries (Marshall et al. 2010).

At this regard, literature shows that for the dentists, a child’s ASD symptoms can interfere with a dental visit and sometimes prevent the dentist from providing the necessary care (Lowe and Jedrychowski 1987). Approximately 60–68% of general dentists reported that they do not treat children with ASD (Dao et al. 2005; Weil and Inglehart 2010). Dental rejection and misrepresentation may be related to dental education, as 58% of parental survey respondents reported that the dentist stated that he/she did not have adequate dental training to treat a child with ASD (Stein et al. 2012).

The reason for the low percentage of general dentists who accept patients with ASD might be related to their set of symptoms.

Firstly, communication issues can affect oral care at home and in the dental office. At home, a lack of verbal communication and understanding can impact on the child necessities, or can lead to problems like self-care, or difficulties with expressing oral pain. In a dentist’s surgery, a lack of communication between patient and doctor can be potentially dangerous when the patient feels pain or is scared and cannot properly communicate this sensation, but might act out as a consequence. (Weil and Inglehart 2010).

Secondly, children with ASD present impairment of social interactions, (Lai et al. 2014). In a dental setting, impairment of social interactions would clearly hinder developing a patient-provider relationship.

Communication can be impaired, and close facial proximity with the doctor whilst the patient is on the dental chair can be uncomfortable.

Furthermore, restricted and repetitive behaviors can become a severe impairment, both for child and for doctor. Visiting the dental office for the first time can be complicated because children with ASD do not like changes to their routine. Moreover, dental procedures are likely to change from one visit to the next. If flossing and brushing is not a part of their daily routine, they might reject it due their aversion to change (Weil et al. 2011). Children with ASD might present alimentary stereotypes that lead to a limitation of what they eat, which can consequently influence their oral care condition.

In addition, children with ASD present the crucial symptom of higher sensory sensitivities. For example, a strong light shining on their face, the flavor of the medical instruments, the contact and the noise of the instrument itself, as well as all the other environmental conditions of the dental office, may represent strong challenges for the patient. (Lai et al. 2012; Fahlvik-Planefeldt 2001; Shapira et al. 1989; American Academy of Pediatric Dentistry 2015; Thomas et al. 2016; Udhya et al. 2014).

Other factors that can also influence their oral health is lack of necessary manual dexterity, thereby resulting in an inability to brush and floss properly (Kamen and Skier 1985). Furthermore, literature shows that children with Autism prefer soft and sweetened foods, and they tend to pouch food inside the mouth instead of swallowing it due to scarce tongue coordination, thereby increasing their susceptibility to caries. Furthermore, some children with ASD are prescribed psychoactive drugs or anticonvulsants, which can cause xerostomia and delayed tooth eruption. They are also more prone to develop bruxism, periodontal disease, and trauma to teeth (Chew et al. 2006). Additionally, due to the frequency of their behavioral disturbances, concerns have increased regarding their ability to access dental care (Lai et al. 2012). Brickhouse et al. (2009) showed that children with ASD who exhibit problem behavior are less likely to have regular dental care. Weil and Inglehart (2012) found a correlation between the severity of ASD-related symptoms in children and their oral health status from the parents’ point of view. Autism is widely recognized as a spectrum, so children with ASD are a heterogeneous group with diverse levels of functioning. Some have the ability to cooperate with dentist during the treatment, (Friedlander 2005; Loo et al. 2009), other can exhibit uncooperative behavior, and this cases are performed with the assistance of advanced behavior techniques such as restraint or pharmacological methods. Stein et al. (2012) and Loo et al. (2009), found that protective stabilization is used in the 20% of dental treatments for children with ASD, significantly greater than that used for TD children (0%), and the use of protective stabilization is the second most used advanced behavior guidance techniques used for children with ASD. General anesthesia is the most frequent behavioral strategy used with children with ASD (Hulland and Sigal 2000) and is utilized from 18% (Marshall et al. 2008), to 37% (Loo et al. 2009; Stein et al. 2012). General anesthesia can be dangerous due to adverse drug interaction (Friedlander et al. 2003) potential injury to the child’s developing brain (Smart 2015), and its high cost may be prohibitive for the families, which can consequently impact upon frequency of dental prophylaxis (Marshall et al. 2008).

All these conditions are experienced by the child’s family as well. The family faces many other barriers to oral health such as the inadequate numbers of dentists that are trained and willing to work with children with ASD (Stiefel 2002; Brickhouse et al. 2009; Weil and Inglehart 2010), and financial issues (Brickhouse et al. 2009; Delli et al. 2013). It has been reported that as few as 10% of practitioners treated children with special needs on a regular basis (Casamassimo et al. 2004; Casamassimo and Seale 2014, 2015).

It is difficult for caregivers to find the right dentist for their child with ASD. Ducker in 2017, shows that this difficulty is subdivided in 4 themes: dentist rejection; dentist misrepresentation, referrals and cost. A study shows that for parents, obtaining access to dental care was more expensive for their child with ASD. Similarly, in 2011 Cidav, Marcus, and Mandell, revealed that families of children with ASD face a significant economic burden. Indeed, on average, mothers of children with ASD earn 35% less than mothers of children with another health limitation and 56% less than mothers of children with no health limitation. They are less likely to be employed and work less hours per week, on average, than mothers of children with no health limitation. On average, children with ASD are less likely to have both parents working. Family earnings of children with ASD are less than those of children with another health limitation and less than those of children with no health limitation. Given the substantial health care expenses associated with ASD, the economic impact of having lower income in addition to these expenses is significant.

Furthermore, the psychological well-being of parents of a child with ASD is significantly influenced by the behavioral difficulties of their child. Existing literature shows higher stress levels, more psychological distress and depressive signs, and increased rates of physical and mental health problems in caregivers of children with ASD (Totsika et al. 2011). Raising a child with ASD can generate stressful conditions which in most cases are associated even with adaptation to child’s routine, interference health care systems and education, coordination of multidisciplinary caretakers, and limited availability of resources (Twoy et al. 2007). Therefore, a scheduled dental visit may represent a stressful event for all parties involved; children with autism, parents and care providers.

Lastly, from a parent’s point of view, the appointment with the dentist is a challenge, which can lead to anxiety and fear for consequences in the dental setting. (Fazli and Kavandi 2015). Those feelings also influence child’s behavior during dental care, leading to a worse child performance and more challenges for the dentist (American Academy of Pediatric Dentistry 2015).

This study seeks to compare the main difficulties experienced by children with ASD and TD, their families and dentists, at home and in the dental environment, in an Italian framework. Other studies have identified the main difficulties of both groups, but has never compared the points of views of both on the same questions, in order to create a clear prospective of the condition.

This study also aims to expand a limited research that can be used to improve patient-centered care and dentist’s approach in comparison with a typical children population.

To our knowledge, this is the first Italian study to approach this subject, while encompassing the points of view of the child, the family and the dentist.

## Methods

### Study Design and Procedure

The study was conducted on two different populations: one is composed of parents of children with ASD and TD children and the other one is composed of dentists. Two different questionnaires were completed by subjects resident in the areas of Chieti-Pescara, Abruzzo Italy. Regarding the questionnaire for the parents, all nursery, elementary and middle schools, rehabilitation centers and social associations within the target areas were asked to join the project. In respect to the questionnaire for the dentists, all hospitals with a dental department and private offices within the target area were asked to join the project. Almost 1000 paper questionnaires were distributed among parents and 100 questionnaires among dentists. Specifically, we reached more than 109 schools and 6 between rehabilitation centers and social associations. Among them 60 schools and 4 between rehabilitation centers and social associations participated. Regarding the dentists, the “Order of medical doctors and dentists” provide us the list of the dentists within the targeted area. More than 100 dentists were reached, and 3 hospitals with the dental department. All 3 hospitals joined the project.

The questionnaires were delivered to the centers that joined the project and then collected. The questionnaires were completed in anonymity.

### Participants

The parents population is composed of parents of children of both sexes, between 3 and 15 years old: 275 parents of TD children, 57 parents of children with ASD. The sample reflect s the epidemiological distribution of ASD in children population (Baio et al. 2018).”

The dentists population is composed of 61 dentists, of both sexes.

### Materials

For the purpose of this study, two multiple choice questionnaires were crafted by authors, one for the parents of children with ASD or TD, and another one for the dentists. The questionnaires were reviewed and edited by a team of expert dentists, developmental psychologists, and statisticians.

Both instruments have been created considering the existing literature and the daily issues experienced by the families and the dentists (Thomas et al. 2016; Lai et al. 2012, Weil and Inglehart 2010, 2012; Stein et al. 2012).

The questionnaires were self-completed by participants. We suppose this detection technique can lead to a higher collaboration with participants, because of the confidential questions and the capacity to collect as much specific information as possible even from other implicated people.

The aim of the “Questionnaire for the parents of children between 3 and 15 years old” is to understand the difficulties faced by parents and children during a dental examination, as well as the barriers to dental care encountered by their children.

The questionnaire is composed by a set of 120 categorical and dichotomous variables, ranging from two to six categories. The questionnaire was mostly designed using frequency scales and Likert scales.

The questionnaire is completed by the parents about their child, with ASD or not. The questionnaire consists of four parts, has a total of 78 questions, and is preceded by a small introduction. In the introduction it is also specified that if the parent has more than one child, the questionnaire should be completed regarding the child with ASD.

The first part (questions 1–17) investigates the sociodemographic characteristics of the child’s family. This part is useful for the detection of the socioeconomic condition of the child and his/her family, and the eventual social or treatment support they have.

The second part (questions 18–46) is about the choices made by the caregiver about the dentist treatment for the child, and about how the child experienced the first time at the dental office.

This information is useful in order to identify child’s and parent’s difficulties before they arrive to the dental office, including child’s fears and the approach that the caregiver have with the dentists, mostly in the autistic case. The aim is to understand how the first visit was, whether (and if so, why) the parent has changed more than one dentist for the child’s treatment, and how the caregiver chooses the medical centers.

The third part (questions 47–66) collects information about the actual dentist chosen by the family, and inquires as to why the caregiver has selected the medical center, how the dentist interacted with the child, and whether this doctor is the one who performed the first visit to the child. This part considers the possible stresses experienced by the caregiver during the dental treatment of the child.

The fourth part (questions 67–78) deals with the oral health habits of the child in everyday life, the relationship of the child with other medical figures, and the possible necessity for better prevention procedures at home. Lastly, parents were asked how important oral health is for them in terms of overall health care.

The “Questionnaire for Dentists” is composed of a set of 64 categorical and dichotomous variables, ranging from two to six categories.

The aim of this questionnaire is to understand the difficulties faced by dentists and children during a dental examination, as well as the barriers to dental care encountered by them and children.

The questionnaire consists of 4 parts, and 68 questions, and was preceded by a small introduction.

In the introduction it was specified that in order to answer questions regarding patients with ASD, parts 1–4 should be completed. Otherwise, for the TD patients, the necessary parts were only 1, 3 and 4.

The first part (questions 1–10), collects professional and demographic information about dentists, and whether they are aware of any local center specifically for the oral health of children with ASD.

The second part (questions 11–39) was completed only by dentists who have patients with ASD, and explores the major difficulties experienced by dentists, children and parents.

These questions investigate the incidence of patients with autism in their surgery, the motivation that brings the caregiver to them, and whether the doctor has any specific skill for the treatment of children with ASD.

This part also thoroughly investigates the child’s first visit to the surgery, and any possible further visits.

The third part (questions 40–60) covers the same topics as the second part, but dentists answer about TD patients instead.

The fourth part (questions 61–67), explores some proposals and opinions that may help to improve the dental treatment experience, for the child and the doctor. The importance of a specific preparation and promotion of prevention procedures at home are also evaluated.

## Results

The data were analyzed with Pycharm (Python language). For both questionnaires the missing values and the overall non-response rate is lower than 5%. Thus, the choice to drop the non-responses, instead of implementing an imputation method, was mostly driven by the low impact of missing values (Agresti 2012).

The methodology adopted, in line with the literature, is grounded on Pearson correlation coefficients, to access the overall correlation among categorical variable, chi square statistics to test independence, odd ratio and logistic regressions to estimate the degree of association and the conditional distributions of the variables in scope. In order to perform logistic regression model estimations, categorical variables were refactored into dichotomous variable.

## Results: Parental Survey

The “Questionnaire for parents of children between 3 and 15 years old”, firstly considered the characteristics of participants. Starting from a description of the respondent’s population, the total population is composed of 332 parents. The mean age of parents of children with ASD is 40.6 and for parents of TD children is 42,7. Regarding the children, TD’s mean age is 9.2 and for children with ASD it is 7.9.

268 respondents took their children at least once to the dentist. Of these, the mean age of parents of TD children is 43.3, while the mean age of parents of children with ASD is 41.9. Among them 18% of parents of TD children and 37% of parents of children with ASD are unemployed. Moreover, 7 out of 59 unemployed respondents have a university degree and more than half (the 69%) of the respondents have a high school diploma or higher.

Most of the respondents have TD children, and more than half of them are female (57%). The mean age of this group is 9.5.

Regarding the children with ASD, the majority of them are male (78%), and the mean age is 8.7.

Secondly, information concerning the motivations and first dental visit were considered. Among the TD children in the sample, 84% have been to the dentist at least one. For the ASD group the proportion is about 74%. The data also shows that the percentage of TD children that goes to the dentist for the first routine dental visit is 85% and 58% for children with ASD.”

Next, more detailed analyses were performed on the data. For the following analysis, only the respondents that took at least ones their children to the dentist were considered (Table 1).

**Table 1:** Contingency table of ASD and attendance to a dental treatment (n = 332)

ASD	No	Yes
Go to dentist		
No	39	25
Yes	203	65

Regarding the different responses to dentist treatments, we were particularly interested in the behaviors of the children at the first dental visit and the general dental practices (Table 2). Data shows that children with ASD present more communication difficulties than TD children, exhibit more aggressive behavior during the dental treatment, are more prone to receive sedation and be rejected by the dentist during the dental practice, and are less likely to follow the home dental prevention rules. Children with ASD are more likely to be treated in public dental surgery than TD children.

**Table 2:** Analysis on first visit child behavior and general dental practices “Questionnaire for the parents of children between 3 and 15 years old” (**p value < 0.1; ***p value < 0.05)

	ASD (%)	TS (%)	F stat	DF	p value
Respondent unemployed	37.1	20.3	9.3	1	> 0.01***
Control visit as the first visit	57.8	85.3	22.9	3	> 0.01***
Child scared by first dental visit	68.9	68.9	24	1	0.88
Child with communication difficulties	53.4	2.5	89.7	1	> 0.01***
Aggressive behavior during first visit	39.6	14.5	15.9	1	0.01***
Rejection of treatment by dentist	12.1	1.3	9.2	1	0.01***
Sedation for relaxing the child during first visit	17.2	7.8	3.8	1	0.05**
Use of technology to relax the child	81	72.3	1.3	1	0.25
Never follow home dental prevention rules	18.9	6.2	10.5	2	p > 0.05***
Pubic dental surgery	36	8	28,96	1	p > 0.001 ***
Parents that changed dentist after the first visit	63.8	61.1	0.04	1	p = 0.84
Respondents consider Dental care highly important	67.2	83.8	9.49	3	p = 0.02***
Need support from dental hygenist and instructor	57.7	66.7	3.6	3	p = 0.3

The purpose of the second analysis was also to describe differences between the 2 samples in terms of different responses derived from the fear of the dentists experienced by the children. The Pearson correlation coefficients for respondent characteristics are summarized in Table 3. Here, it is shown that the strongest correlation is between children with ASD’s fear of the dentist and difficulties in dental treatment.

**Table 3:** Pearson correlation coefficient for selected metrics from “Questionnaire for the parents of children between 3 and 15 years old”

	Parent’s fear	ASD Child	Sex	Age	Fail fo the first visit	Child Aggressive behavior during first visit	Child fear of the first dental visit
ASD Child	− 0.07						
Sex	0.06	− 0.29					
Age	− 0.07	− 0.14	0.09				
Fail fo the first visit	0–03	0.13	− 0.05	− 0.15			
Child Aggressive behavior during first visit	0.04	0.27	− 0.04	− 0.11	0.02		
Child’s fear of the first dental visit	0.19	0.01	0.13	− 0.12	− 0.06	0.32	
Child Communication difficulties	− 0.04	0.5	− 0.07	− 0.19	0.27	0.21	− 0.02

The relationship between respondent’s fear of the dentist and the children’s fear is subsequently investigated, in order to access whether the transmission of fear from parent to child is different between ASD and TD. It is then considered whether this fear becomes an obstacle to medical treatment and to the returning rate. Starting from parent’s fear of the dentist and children’s fear, the contingency table shows an evident relation between the transmission of fear from parents to children, with a statistically significant marginal odd ratio of 4.53. The degree of association between these two dimensions, in our sample, is very high: the estimated odd of child fear, for children with parent that are scared by the dentists, is 4 times greater than the estimated odd for child with parents that have no fear of it. The estimated chi square statistics support our result, rejecting the null hypothesis of independence among child fear and respondent fear (Table 4).

**Table 4:** Contingency table of fear of the dentist of the interviewed and fear of the dentist of the children (n = 251)

	Child’s fear	No	Yes
Parent’s fear	ASD		
No	No	55	99
	Yes	18	33
Yes	No	5	34
	Yes	0	7

Marginal Odd ratio: 4.53 (p = 0.002), CI [1.72,11.96]

Furthermore, parents fear increases by 1.5 the probability of having a child that have fear of the dentist. There is no evidence that the association differs among control and case groups (Table 5).

**Table 5:** Logit model for child fear of the dentist over Parents fear of the dentist

Variables	Coef	Std. err	p value
Parent’s fear of the dentist	1.5216	0.4968	0.0022
ASD children’s fear	0.0994	0.3298	0.7632

Pseudo R-squared: 0.040

No. Observations: 251

The success or failure of the dental treatment, in relation to the fear of the dentist, is subsequently investigated.

Data in Tables 6 and 7 show that despite the small number of respondents for which the first visit to the dentist was ineffective, the combination of child fear of the dentist and ASD highly affects the success of the visit, as shown by the positive and statistically significant coefficients of the logit model. Indeed, if 100% of children with ASD and no fear of the dentist attended the visits, 35% of children with ASD and fear failed the visit.

**Table 6:** Contingency table of fear of the dentist of the interviewed and fear of the dentist of the children

	First visit fail	No	Yes
ASD	Child’s fear		
No	No	59	1
	Yes	131	2
Yes	No	18	0
	Yes	26	14

Marginal Odd ratio: 7.8 (p = 0.047), CI [1,60.2]

**Table 7:** Logit model on first visit fail over child fear

Variables	Coef	Std. err	p value
Child’s fear of the dentist	2.1820	1.0626	0.0400
ASD children	3.0007	0.6649	0.0000

Pseudo R-squared: 0.283

No. Observations: 254

We investigated the reasons why a dentist may fail to conclude a dental treatment. There may be several, however verbal inability to express pain could be a concrete obstacle to treatment success. Data reported in Table 8 shows that difficulties in verbal communication play a role in predicting aggressive behavior, with a statistically significant marginal odd ratio of 4.7. However, grouping the observations in ASD and TD children, the combination of ASD and verbal difficulties have an odd ratio of 3, with more than a half of children with ASD and verbal difficulties having aggressive behavior during the visit.

**Table 8:** Contingency table of communication difficulties and aggressive behaviors

	Aggressive behavior	No	Yes
ASD	Communication difficulty		
No	No	161	27
	Yes	4	1
Yes	No	20	7
	Yes	15	16

Marginal Odd ratio: 4.7 (p < 0.01), CI [2.2,10.1]

Conditional Odd ratio (ADS = 0) = 1.49 (p = 0.73), CI [0.16,13.8]

Conditional Odd ratio (ADS = 1) = 3.0 (p = 0.049), CI [1, 9.3]

This is not true for TD group were the conditional odd ratio is closer to the unit level, suggesting lower association between the two variables. Trying to capture the magnitude of the association, the marginal effect of each dimension in predicting aggressivity, the estimate coefficients of the logit model show a positive and significant association with aggressive behavior (Table 9). It is worth noting that the adjusted R square is very low, meaning that the predictors do not capture the determinant factors influencing aggressive behaviors, especially for the TD group. In the model for child’s fear, the percentage of explained variance increases, associating a positive and significant coefficient also to the child’s fear of the dentist (Table 10).

**Table 9:** Logit model for communication difficulties of children with ASD and aggressive behavior

Variables	Coef	Std. err	p value
Communication difficulties	0.9566	0.4885	0.0502
ASD children	0.8511	0.4344	0.0501

No. Observations: 251

Pseudo R-squared: 0.077

**Table 10:** Logit model for communication difficulties, fear of the dentist and aggressive behavior

Variables	Coef	Std. err	p value
Communication difficulties	1.0246	0.5638	0.0692
ASD children	1.0367	0.4851	0.0326
Child’s fear of the dentist	3.6438	1.0359	0.0004

Pseudo R-squared: 0.221

No. Observations: 251

Finally, prevention procedures regarding both groups are discussed.

It seems that, more than half of parents of both groups (57% for ADS and 66% for TD), declared that they need support from the dental hygienist and instructions for a proper oral hygiene. On the other hand, regarding the importance of dental health for the well-being and health of the body, 67% of respondent parents of children with ASD consider oral and dental care of great importance, and more than 83% of respondents with TD children express the same concern.

Almost all of the ASD sample claimed to have the same difficulty during other medical visits.

## Results: Dental Practitioner Survey

In the “Questionnaire for Dentists”, characteristics of participants are considered. The aim is to better understand the dentist’s point of view regarding the child’s behavior, and whether the dentist deliberately adapts his behavior according to the different patients treated. Therefore, in this case, the sample was not stratified into control and treatment groups, but rather to each respondent’s answers to questions related to ADS or TD patients.

Starting from a description of the respondents’ population, the sample is composed by a very low percentage (10%) of dentists specialized in pediatric dentistry. The large majority of dentists (69%) work mainly in private offices, and more than half (68%) of them are males. Mean age of the sample population is 40.2.

In the sample only 39 dentists have at least one patient with ASD. Their mean age is 43.2. Of these, almost half (46%) claimed to have at least some difficulties in treating children with ASD. Mean age of the respondents who have never treated children with ASD 32.4.

Information regarding the motivations and first dental visit were then considered. Data shows that the percentage of TD children (51%) who have been to the dentist for the first routine dental visit is slightly higher than the percentage of children with ASD (46%).

Next, more detailed analyses were performed on the data. The aim of the following analysis is to identify differences between TD children and children with ASD, in terms of different responses to dentist behaviors or competences.

Pearson correlation coefficients for a selected set of dichotomous variables related to ASD behavior are shown in Table 11.

**Table 11:** Pearson correlation coefficient for selected metrics from “Questionnaire for the dentists” (*p value > 0.01 and < 0.05; **p value < 0.01 and > 0.001; ***p value < 0.001)

	Works in Private sector	Treated ASD Child	Specific course on ASD	ASD child fear of the armchair	ASD child fear of the light	ASD child fear of the noise	ASD child fear of the mask	ASD child fear of the Flavour	ASD child fear of the dentist
Treated ASD Childs	0.1								
Specific course on ASD	0.13	0.32*							
ASD child fear of the armchair	− 0.17	0.3	0.03						
ASD child fear of the light	− 0.16	0.22	0.18	0.33*					
ASD child fear of the noise	0.1	0.55*	0.41*	− 0.05	0.4				
ASD child fear of the mask	0	0.27	0.08	− 0.23	− 0.03	0.31*			
ASD child fear of the Flavour	0.13	0.08	− 0.07	0.07	− 0.05	0.14	0.29		
ASD child fear of the dentist	0	0.22	0.21	0.19	− 0.04	0.12	0.15	− 0.23	
Sedation for relaxing child	0.12	− 0.53*	− 0.24	− 0.15	− 0.1	− 0.21	− 0.12	− 0.07	− 0.35

It is necessary to underline that although the number of observations is limited, and therefore the significance of the correlation poorly fulfill the limit of 0.05 significance level, some interesting results can be discussed. The first evidence is related to the positive correlation between the attendance of courses about autism for dentists, and the treatment of children with ASD. This is due to the fact that 30% of dentists who treated children with ASD attended a specialistic course on the topic.

Moreover, a higher percentage of dentists (41%) who treat patients with ASD claimed that parents of children with ASD are stressed by the dental visit of their children versus a slightly lower percentage (39%) of the dentist declared that parents with TD children are stressed by the dental visit of their children.

With regard to the reason for the stress experienced by children with ASD during the dental treatment, more than half (67%) of the dentists who treat children with ASD claimed that patients with ASD are scared by noises, followed by the chair (30%), the mask (25%), and by the light (17%). Comparing those results with the TD population, a lower percentage of dentist (25%) declare that TD children are scared mostly, by the chair (54%), the light, (31%), the mask (11%), and the noises (4%).

Relaxation strategies used by the dentists during the first dental visit are also considered in our analysis. Data shows that a low percentage of them use sedation. Where sedation is used, a higher percentage of dentists claimed to administer sedation more often with children with ASD (10%) than TD children (8%).

Data also shows that the most common methods for helping children with ASD to relax is the use of toys, as declared by almost half of the dentist population. This is followed by the presence of the parent during the dental surgery, the use of tablets or other technological devices, and lastly by coercive methods. With respect to TD children, the most common methods are the use of toys, as declared by almost half of the dentist population (52%), followed by the involving of the parents, use of tablets and other devices, and coercive methods.

Where prevention is concerned, almost half of the sample declares that they strongly support the need of a better oral care prevention system and believe that specific training is necessary in order to better treat patients with ASD.

## Discussion

In order to understand the difficulties experienced by both populations, this study includes two groups of respondents, parents and dentists.

These qualitative findings show some disparities between the parents of children with ASD, and parents of TD children.

The parents of children with ASD are more likely to be unemployed than the parents of TD children. This condition is confirmed in literature (Cidav et al. 2012) and demonstrates the strong social impact of those syndromes, and the consequent disability of the families. Parental unemployment and the chronic disability of the child can contribute to lower accessibility to private dental offices, higher presence of oral pathologies, and higher feeling of fear and concern experienced by parents during the child’s oral health care. This condition is confirmed by the theory of the “Inverse Care Law” (Hart 1971). Our findings show that for parents of children with ASD, oral care is less important than for parents of TD children. Following Hart’s theory, public health interventions aimed at controlling diseases or risk factors and providing new and more efficient services, while being addressed to the general population, are not uniformly distributed within it. The first to enjoy the effects are the wealthy, to whom a higher cultural level allows messages to be received and opportunities to be seized, while the poorer social classes, despite having the same opportunities to take advantage of these services, do not. The final result is an increase in inequalities.

The reasons of the inverse care law are comorbidity and fatalism. In our case, the first points out that for the families of children with ASD, the oral care of their child is not an important issue. In fact, in addition to the possible presence of other pathologies in the family, the other important factors like life habits, economic problems, psychological stress and working conditions could prevent parents from realizing the severity of their children’s oral health problem. Literature shows that these children are the main users of the emergency services and the last users of the public care and prevention services (Hausen 1997; Vohra et al. 2016).

Fatalism, on the other hand, is the belief that we can do nothing to change the state in which we find ourselves. To solve the problems related to fatalism and comorbidity a better quality of life, economic stability and more culture would be needed, the latter not being understood only in terms of “health literacy”, as greater knowledge of medical problems, but precisely as a level of education in general (Grant 2016).

Improving dental care prevention, to the poorer social classes, can avoid the possibility to experiencing the firsts dental visit in a condition of emergency, pain and stress.

Indeed, a dental visit is an essential instrument for the detection of important systemic diseases, and is fundamental for the general health (Minister of Health 2017).

The approach of the child at first dental visit is fundamental for a good diagnosis, health care (Isong et al. 2014; Bezabih et al. 2013), psychological wellbeing of the child at future visits and with other health care providers (Koneru and Sigal 2009).

Reasons for making an appointment with the dentist are important predictors of the approach of the child and the family to the dental visit. Data from the questionnaire identify two main reasons for making an appointment with the dentist: specific pathologies, even linked with ASD, and first routine dental visit. The last is more common between the TD children population, supporting the hypothesis of lower prevention habits between the ASD population.

Another important factor that emerges from our data regarding the approach to the dental visit, is the eventual fear of the dentist experienced by the parents. The data shows that this fear is transmitted by parents to the children. If parents experience stress and are scared during the dental visit, this feeling may be transmitted to the child. This is true for both ASD and TD populations. (Klingberg et al. 1995; Klingberg and Broberg 2007; Bezabih et al. 2013; Lara et al. 2012). It may be possible that parents in addition to their own fear of the dentist, during dental examination of their children are even more stressed and scared.

Regarding parents of children with ASD, they are aware of their children’s difficulties and daily disability, or they may be victims of dental or medical negative experiences with their children, or frightened by the reactions that the child might have or by the fear that the child suffers. It is common for parents of children suffering from disorders to go through a state of anxiety and concern, during the dental examination of their child (Fazli and Kavandi 2015). Furthermore, dentists in our sample claim that almost half of the parents of children with ASD experience high levels of stress during the dental visit of their child. This would support a hypothesis that the parents are scared by the possible reactions of their children, or by the negative outcome of the dental visit and treatment. This is may be due to previous experience or the scarce experience they have with medical treatments. In fact, parents have an essential role in guiding children behavior, in both groups, corroborating the idea that a previous meeting with the dentist’s equipe and deep prevention approach, may be fundamental for the children’s and parents’ health care.

A further important factor is the serious difficulty in communication of the children. Data from the study highlights that children with ASD present a deficit in communication behaviors and this difficulty predicts the expression of aggressive behavior towards the dentist, maybe due to greater state of stress of the child during the visit (Sadiq et al. 2012). In order to achieve a higher probability of a successful visit, it would seem necessary that both the dental equipe and children should have the instruments to communicate with each other since the first visit to the dental surgery. Furthermore, the sample shows that a low percentage of dentists in the territory has a specialization in pediatric dentistry, and some of them have attended courses about the treatment of children with ASD. These dentists are more prone to treat children with ASD, supporting the hypothesis that a deeper knowledge of the argument can support the acceptance and the treatment of those patients.

The second step of a dental visit is the dental treatment. During the treatment, different behaviors and emotions can emerge. One of the aims of this study was to investigate about the possible causes of fear. Data from the instrument shows that both groups of children, with ASD and TD, are scared of going to the dentist. Furthermore, the group of children with ASD presents a higher percentage of children scared by dental visit, and a lower percentage of children who have been at least one time at the dental office. It may be possible that lower accessibility to the dental surgery can lead to a higher fear experienced during the first dental treatment, due for example to less habituation process, or less possibility to find an appropriate medical doctor, or less attention to oral health, or because of the possible behavioral reactions of the children with ASD. Indeed, data show that children with ASD have more fear of the dentists, and this lead to more difficulties during the dental treatment. Fear of the dentist is a strong component for a failed visit. The results demonstrate that fear of the dentists experienced by children with ASD often leads to a failed visit. We can deduce that the combination of the two factors plays a crucial role in identifying dentist treatments’ attendance. Indeed, the outcome of the first dental visit is fundamental, in order to avoid scaring the child and leaving them with a memory of fear.

Even the environment of the dental office aggravates the condition of fear and anxiety already experienced by the child. (Fahlvik-Planefeldt and Herrstrom 2001; Lai et al. 2012; Shapira et al. 1989; American Academy of Pediatric Dentistry 2015; Thomas et al. 2016; Udhya et al. 2014). The stressful environment can push the child to uncooperative and aggressive behaviors or complaints, which results in wasted time for dentists and a failing dental treatment for the child. Children with ASD are mostly scared by the noises, by the dentist’s chair, the mask worn by the dentist and the bright light on their face. TD children are also scared by the dental instruments, but with less impact and by different type of instruments. These factors are important stressors for the child, in particular for the child with ASD, due to the excessive sensorial stimulation of the environment (Wibisono et al. 2016). The literature shows that the fear of suffering and of the environment experienced by the child, can lead to aggressive behavior (Shapira et al. 1989). Our data confirms the more common aggressive behavior of children with ASD, than TD children. Supporting this view, dentists’ population declare that out of the dentists who treat patients with ASD, almost half of them encounter difficulties in treating children with ASD. They also attest that even at the second appointment, children are scared and frightened, meaning that the psychological prime obtained by the first visit is difficult to erase.

How do dentists manage the autistic behaviors in order to perform the treatment?

Our data shows that some doctors reject children with ASD because of these behaviors, or reject those children a priori.

On the other hand, dentists carry out a lot of unusual strategies in order to perform the dental visit (Goldsmith and Leblanc 2004), like toys or technological instruments (tablet or computer) or the presence of parents during the treatment, or coercive methods. However, the parents’ sample declares that dentists are more prone to use drugs and physical constraints with children with ASD, than with TD children. Furthermore, a significant percentage of children with ASD are treated in public hospitals, maybe because it is easier to administer sedation to them there (Youan-Yuan and Wei 2013). On the other hand, our study shows that a higher percentage of parents of children with ASD turn to public services, than parents of TD children, but a small percentage of them have received sedation. Taking into consideration the demographic conditions of our sample, (schooling and unemployment) it may be possible that the choice of a public service is essentially economic—a condition confirmed by Duker et al. (2017), who writes that parents noted that obtaining access to dental care is more expensive for their children with ASD; otherwise, this choice might be due to the higher percentage of dentists specialized in neurodevelopment disabilities.

The dentists’ sample shows that it is common to abandon treatment part-way through an appointment due to the child’s stress levels – both in the case of ASD and TD. The dentists reveal that in order to relax and consequently treat the child, the majority of the sample does not use anesthesia or drugs. This may be because the majority of dentists of the sample work only in private sector, where administering anesthesia is less common.

The sample also shows that families with children with ASD are slightly more prone to change dentist from the one who has performed the first visit than families with TD children. It may be that both families and dentists recognize the great difficulty of the treatment of a child with ASD just after the first dental examination. Subsequently, the families have to find an expert dentist (Udhya et al. 2014; Friedlander 2005).

During this process the child has already acquired a negative and potentially scarring experience from the first dental visit.

The questionnaire is also centered on prevention conditions of our sample, in order to create an adequate pattern of approach and better comprehend the most common medical conditions of the patients. Literature shows that oral health care prevention is an important factor for oral health and that is more complicated for the families of children with ASD (Rowan-Leg and Canadian Paediatric Society, Community Paediatric Committee 2013; Anderson et al. 2017). The data shows a positive attitude towards oral health care prevention for both groups, even if the percentage of children with ASD who have never followed home dental prevention rules is higher. Therefore, the real difference between the two groups may be attributed to the quality of the prevention, rather than to the quantity. It is necessary to consider the overall condition of disability and difficulty of health care, experienced by children with ASD. On the other hand, the complications experienced by parents of the two groups of children during oral health care prevention are different. Indeed, the questionnaire inquires into the propensity of the parents to be helped and supported by an expert during the learning prevention process. Data shows that parents of children with ASD claim to be more in need of support than parents of TD children, perhaps due to the deficits of the child and the lack of medical support.

With regard to the prevention condition as seen by doctors, the community claims the necessity for a better oral health care prevention for children, and for an adequate training to parents by doctors.

Doctors support the need of a specific training and education for the treatment of children with ASD, or at least the presence of an expert doctor in the dental office.

Data shows the need for a specific approach to the patient with ASD.

Both doctors and parents should have the opportunity to learn how to deal with the child during the visit. Parents complain about the difficulties experienced during all types of medical visits. This data is not confirmed by the sample of parents of TD children.

Autism is a chronic condition which presents a complex medical and psychological pattern that involves the patient and his family (Kemper and Bauman 1998). The treatment of a pediatric chronic patient has to comprehend a multispecialistic and integrated approach for his medical, psychological and social wellbeing. For this purpose, the collaboration of parents and medical team is vital. “Health literacy” among the patient and family is equally as important, in order to reach a higher level of patient empowerment, which is necessary for the family and patient’s self-management. The treatment of pediatric psychiatric patients has to be managed by a multisectoral intervention, starting from specific university classes for all the medical equipment. This necessity is confirmed by our data, from a medical and parental point of view. These classes may help the doctor to understand the symptomatology of this psychiatric disease, and learn how to approach, communicate with and treat the child. At the same time, the medical equipment has to be a reference for the parents regarding the health care prevention and the dentist appointment. Weil and Inglehart (2010), shows that the better prepared the dentists were by their dental education about the treatment of patients with special needs, the more positive their attitudes were.

The goal is to give parents the chance to turn to an expert doctor from the first visit, and make the dental care experience a positive one for parents, children and doctors.

This may reduce the chances of needing to change doctor after the first visit, reduce the use of drugs and the child’s aggressive behaviors, and treat the child in the best way possible.

## Conclusion

The dental care experiences of families of children with ASD and dental professionals that treat this population are essential to determine best practices to improve access and develop interventions. By better understanding the barriers to dental care the ASD population faces, dentists and other health care providers can work to minimize difficulties encountered by children with ASD. In this study, parents provided information about their children’s dental care experience and dentists about their patients. As emerged from the study, children with ASD present difficulties with the dental health care process, resulting in a lower probability of effective treatment.

On the other hand, dentists are faced with an unknown disorder, as autism often is for them, and claim a deep knowledge in order to treat the child as best as possible.

The comparison and the use of this information can provide care and alleviate some of the challenges experienced by these two populations, whilst potentially improving health care. This study may help to identify the needs of the populations and design new methods and procedures to best serve children with ASD and develop collaboration between families, and all health care providers.

## Limitations

This study has potential limitations. First, the limited samples of dentists and families with children with ASD. Second, the different levels of gravity of ASD symptomatology were not taken in account and at last the territory of research was restricted.

## Implications for Further Research

The data shows how dentists and parents can use multimedia tools such as tablets or smartphones to calm children with ASD and treat them with greater ease and effectiveness. Future studies should concentrate on the developing of these tools. In fact, these multimedia tools may be used as assistive technologies by parents and dentists for children with ASD and TD, through interactive games thought to be suited for children with ASD. These multimedia tools are widely used by our children’s generation, the Millenial generation, and children with ASD show a strong propensity to use them (Dettmer et al. 2000; Goldsmith and Leblanc 2004; Griffiths 2002; Mazurek et al. 2012).

Furthermore, these tools are used daily by caregivers of any social class and literacy (Olson et al. 2011). The aim should be helping the children to sensitize with the dental visit, the dentists to discover what kind of difficulties the patients might present, and how to deal with them.
